# Variation in Spatial Access to Substance Use Treatment for Older Adults: A Nationwide and Kansas-Specific Analysis

**DOI:** 10.1093/geroni/igaf122.2515

**Published:** 2025-12-31

**Authors:** Elizabeth Bambury, J Tom Mueller, Elizabeth Ablah, Sharon Fitzgerald Wolff

**Affiliations:** University of Kansas Medical Center, Andover, Massachusetts, United States; University of Kansas Medical Center, Kansas City, Kansas, United States; University of Kansas Medical Center, Kansas City, Kansas, United States; University of Kansas Medical Center, Kansas City, Kansas, United States

## Abstract

Substance use disorder is often overlooked and undertreated among older adults – a growing population especially in rural America. Treatment programs specifically tailored to meet their age-related needs can improve recovery outcomes. This study examines spatial access to these specialized substance use treatment (SUT) programs both nation-wide and with a detailed focus on the state of Kansas. We obtain data on SUT facilities from the 2022 National Directory of Drug and Alcohol Abuse Treatment Facilities. Distances between census tract centroids and facility locations were measured, and spatial accessibility was assessed using a two-step floating catchment area method to generate a spatial access index (SPAI). For older Kansans, a second SPAI using driving distances was computed. Results are displayed nationally and for the state of Kansas, and also stratified by rurality using Rural-Urban Commuting Area Codes. Of 12,102 identified SUT facilities, 3,614 (29.9%) offered programs for older adults. Among 84,120 census tracts, 4,323 had no such facility within 50 miles, affecting 2,589,240 adults aged 65 and older. On average, the nearest older adult program was 13 miles away (SD = 19), with small rural tracts averaging 34 miles farther than urban tracts (p < 0.001). SPAI values further highlighted disparities in rural areas, even after accounting for service demand. Our findings reveal that for millions of older adults, particularly in rural areas, distance poses a significant barrier to substance use treatment. Expanding access to specialized treatment programs in underserved areas is essential to promote healthier aging among older adults.

